# ‘They only smoke in the house when I’m not in’: understanding the limited effectiveness of a smoke-free homes intervention

**DOI:** 10.1093/pubmed/fdaa042

**Published:** 2020-04-23

**Authors:** R O’Donnell, A Amos, S W Turner, L Adams, T Henderson, S Lyttle, S Mitchell, S Semple

**Affiliations:** 1 Institute for Social Marketing and Health, Faculty of Health Sciences and Sport, University of Stirling, Stirling FK9 4LA, UK; 2 Usher Institute, University of Edinburgh, Edinburgh EH8 9AG, UK; 3 Institute of Applied Health Sciences, University of Aberdeen, Aberdeen AB25 2ZD, UK; 4 NHS Lanarkshire, Airdrie ML6 6DB, UK; 5 NHS Lanarkshire, Hamilton ML3 0TA, UK

**Keywords:** children, education, gender, intervention, qualitative, second-hand smoke

## Abstract

**Background:**

Children’s second-hand smoke (SHS) exposure in the home is highest in socio-economically disadvantaged areas. Personalized household air-quality measurements can promote changes in smoking that reduce SHS exposure. The ‘First Steps 2 Smoke-free’ (FS2SF) intervention is the first to trial this approach delivered as part of health professionals’ routine work. This paper reports the findings of qualitative interviews with participants that explored their experiences of the intervention and why outcomes varied.

**Methods:**

120 women were recruited from the NHS First Steps Programme, which supports disadvantaged mothers. They received either personalized feedback on their home air quality and advice on reducing SHS or standard SHS advice. Qualitative interviews with 15 mothers were analyzed thematically using the Capability, Opportunity, Motivation, Behaviour (COM-B) model.

**Results:**

The intervention increased women’s capability to change home-smoking behaviour, through increasing awareness and salience of SHS risks to their children, and motivation to act. However, taking effective action was constrained by their limited social and environmental opportunities, including others’ smoking in the home.

**Conclusions:**

The FS2SF intervention was ineffective as it was unable to fully address the precarious, complex life circumstances that make creating a smoke-free home particularly difficult for women experiencing intersecting dimensions of disadvantage.

## Introduction

Exposure to second-hand smoke (SHS) in the home causes adverse health outcomes in children and adults.^1^^,^^2^^,^^3^ The proportion of children exposed to SHS in the home has declined in the UK, but there is continuing inequality in exposure levels.^4^^,^^5^ In Scotland, under 1% of children in the most affluent areas report regular SHS home exposure, compared to 15% of childrenje in the most deprived areas.^5^ Disadvantaged parents can face challenges in creating and maintaining a smoke-free home, particularly when sole caring for young children in accommodation with limited access to suitable space to smoke outside.^6^^,^^7^^,^^8^^,^^9^

A range of interventions have been developed to promote smoke-free homes, for example using counselling approaches, feedback of a biological measure of children’s SHS exposure, school-based strategies, nicotine-replacement therapy and educational materials. However, reviews of interventions to reduce SHS in households with children have concluded that there is insufficient evidence to recommend any specific approach.^10^^,^^11^^,^^12^

Two British studies using personalized measurements of air quality in disadvantaged households have shown promise. The REFRESH ^13^ feasibility study involved smoking mothers in Scotland. It compared changes in home air quality, measured by airborne concentrations of fine particulate matter < 2.5 μm in size (PM_2.5_) and smoking in the homes of mothers who received personalized home air-quality data as part of a motivational interview with those who received only the motivational interview. REFRESH showed reductions in PM_2.5_ levels, with women motivated to protect their children because of their new knowledge about their home SHS levels.^13^^,^^14^ The second intervention^15^^,^^16^ used a similar approach, but included nicotine replacement therapy (NRT) for temporary abstinence and involved women with children under 5 years from socio-economically disadvantaged communities in England. This RCT found significant reductions in the intervention compared to the control group in home SHS levels at 12 weeks follow-up.^16^

In both studies, women reported that personalized feedback was the key motivating factor for changing smoking in the home. However, these interventions used labour-intensive recruitment^16^ and delivery methods,^13^ involving staff with dedicated time for delivery. The feasibility of health professionals delivering similar interventions as part of routine practice remains uncertain, particularly given declining resources for tobacco-control work.^17^

The NHS Lanarkshire First Steps Programme (FSP) provides home-based, one-to-one support for vulnerable women in socio-economically disadvantaged areas of South Lanarkshire. It works with mothers of pre-school children and pregnant women to develop parenting skills and encourage play and healthy lifestyles. At the time of the study, over 30% of clients smoked with 48% of homes having one or more smoking-adult resident. The ‘First Steps to Smoke Free’ (FS2SF) study aimed to test whether personalized air-quality feedback could be delivered by First Steps (FS) workers as part of their routine work and whether this reduced home SHS levels. An asset-based approach guided study development, considering women’s own resources, networks and skills alongside their needs.^18^^,^^19^ The FS2SF quantitative results are reported elsewhere.^20^ In summary, there was no statistical difference between the standard and enhanced intervention groups’ 1- and 6-month PM_2.5_ measurements. To understand why FS2SF had no effect, this paper reports the findings of qualitative interviews with women who received the enhanced intervention, which explored their experiences of the intervention and why behaviour change outcomes varied.

## Methods

### Study sample

FSP clients who were over 16 and smoked or lived with smokers could participate. FS workers invited eligible women to participate in the study, provided information sheets and gained written informed consent. Project home visits were built into the existing weekly visits. Full engagement over the 6-month intervention period involved nine visits. 171 women were invited to take part, of which 120 agreed (response rate 70.2%).

### Design

**Fig. 1:**
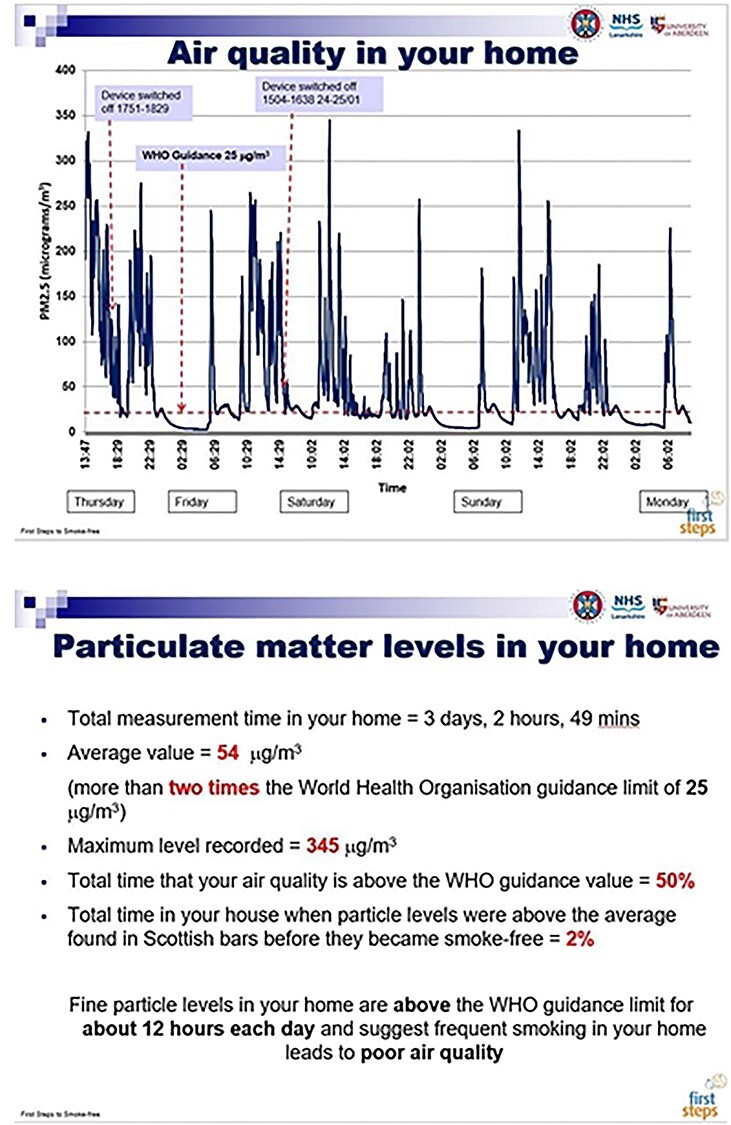
Example of the air-quality feedback graph and summary quantitative information on home air-quality measurements given to the participants.

Seventeen FS workers received a half-day course on the health effects of SHS, using the air-quality monitor and discussing the measurements with participants to encourage them to make their homes smoke free. Participants were randomized to Group A or B. A Dylos DC1700 air-quality monitor was installed in the home by the participant’s FS workers to measure PM_2.5_ for 3–7 days at baseline, +1 month and +6 months. An FS administrator downloaded data from air-quality instruments and prepared personalized feedback graphs. Group A received standard NHS Lanarkshire advice on the harmful effects of SHS after the baseline visit. Group B received standard advice plus personalized air-quality feedback at the baseline, +1 month and + 6 month measurements. Fig. 1 shows a hypothetical example of the air-quality feedback graph and information on home air quality that Group B participants received. Questionnaires assessed smoking and household rules at baseline, 1- and 6-month follow-up.

### Qualitative interviews

On completing the 6-month visit, 20 purposively selected Group B participants were invited by their FS worker to participate in an individual interview in their home, with one of the authors (RO). Participant selection aimed to optimize diversity in home smoking rules, change achieved, household circumstances, seasonality and FS workers involved. Two participants declined to take part in an interview and three participants agreed to take part but were uncontactable when the researcher attempted to schedule their interview. It was not possible to identify the characteristics of these five participants, due to confidentiality issues. Data collection and thematic analysis were conducted in parallel. Data saturation was reached, with no new themes emerging, by the 15th interview. The semi-structured interviews were audio-recorded with participant consent and lasted approximately 45 minutes. The interviews explored motivators and barriers to changing smoking behaviours, knowledge about SHS and health risks, perceived utility of air-quality measurements and intervention engagement. Participants received a £10 voucher at baseline and a £20 voucher at the 6-month visit. Interviewees received a further £10 voucher. Ethical approval was obtained from the NHS North of Scotland Research Ethics Committee.

### Qualitative analysis

**Fig. 2:**
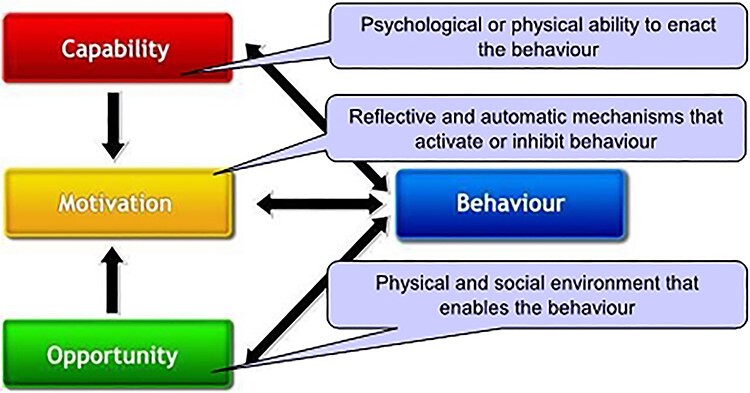
The COM-B System - a framework for understanding behaviour change.

Interviews were verbatim transcribed. Initial coding was deductive, with codes derived from topic guide headings. Two authors (R.O., A.A.) independently read each transcript, noting topics and themes and potential connections between them. Deductive codes were then supplemented with more inductive codes derived from emergent themes in the data.^21^ Emerging themes provided a coherent fit with the COM-B model,^22^ which proposes that for individuals to engage in behaviour change, they must have the ‘capability’, ‘opportunity’ and ‘motivation’ (Fig. 2). The COM-B model was chosen as an appropriate analytical theoretical framework, because it considers both individual-level constructs (capability and motivation) and wider social contexts (opportunity). Relevant themes were organized by each of the three COM-B constructs. Discrepancies regarding theme or interpretation were discussed and resolved by two members of the study team (R.O., A.A.).

## Results

### Capability - knowledge, awareness and communication

**Fig. 3:**
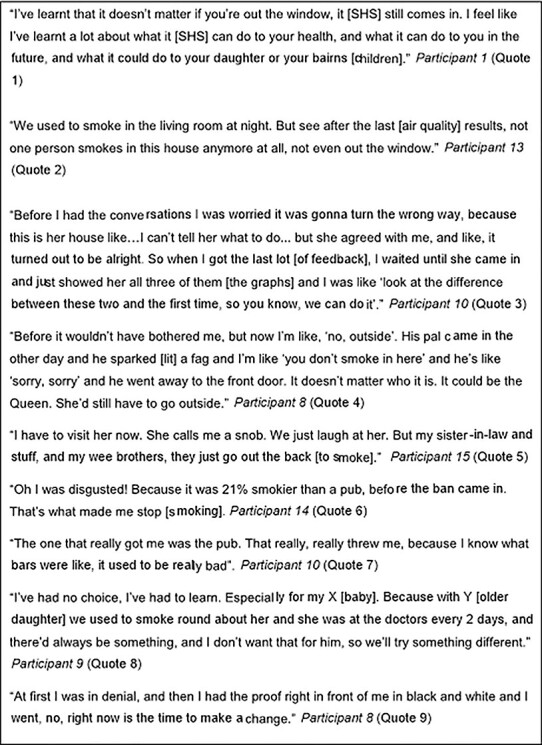
Increased capability and motivation to create a smoke-free home.

Interview findings indicated that participants found the intervention acceptable and understood the data they were shown, in some cases with assistance from their FS worker. Fourteen reported increased capability to change their smoking through increased knowledge and/or awareness about the health risks to children of SHS in the home, and how SHS remains suspended in the air (Fig. 3 quote 1). Some felt that having personalized results helped ‘prove’ facts about SHS that they had previously not believed. Participants also talked about their awareness of the ineffectiveness of previous strategies to reduce their children’s SHS exposure (i.e. opening a window and/or smoking in one room). During the intervention, they had adopted what they perceived to be the more effective strategies, from standing out on the veranda to smoke with the door shut to quitting smoking (Fig. 3 quote 2).

**Fig. 4:**
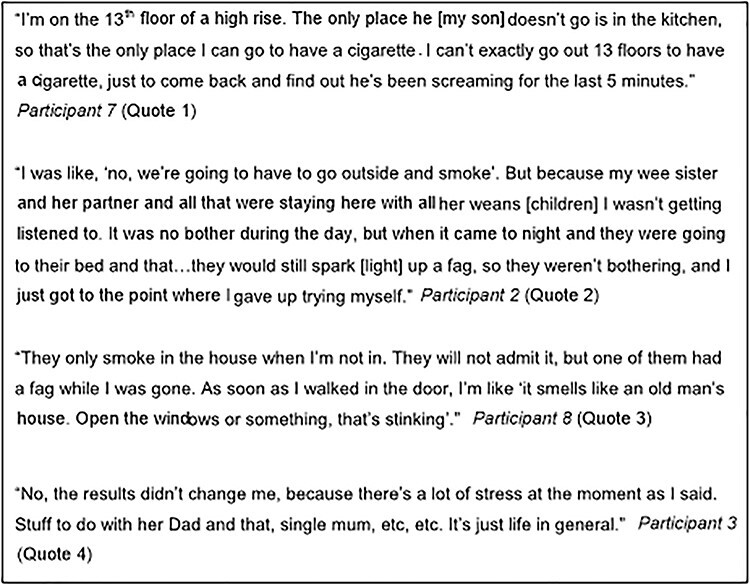
Opportunity to create a smoke-free home 209 × 297 mm.

Most participants said they had talked with family and friends about the intervention, passing on their knowledge about SHS levels in their home. Several spoke with family members and/or regular visitors to gain their assistance in creating a smoke-free home. One woman, a non-smoker living with her baby and her mother (a smoker), had raised the issue with confidence using her personalized feedback (Fig. 3 quote 3). Subsequently, her mother decided to smoke outside the back door, with the door shut. Another participant spoke about changing her attitude toward visitors smoking in the home (Fig. 3 quote 4). However, a few participants described unsuccessful attempts to get others on board with new household smoking rules. Participant 15 described how her mother had been unhappy about having to smoke outside when she visited (Fig. 3 quote 5). These experiences highlighted the importance of psychological strength and stamina as a facet of capability related to persuading others in the household to support creating a smoke-free home.

### Motivation - automatic (emotion-based) and reflective (belief/intention-based)

Thirteen participants reported increased motivation to change their smoking, with most expressing shock that their personalized feedback displayed higher PM_2.5_ readings than expected. Feelings of shock that were often discussed in relation to specific feedback on the percentage of time measurements were above the concentrations of SHS in Scottish bars prior to smoke-free legislation (Fig. 3 quote 6). This was surprising as smoke-free bars were implemented in 2006, but participants often had vivid images of their ‘smokiness’, which was more salient than the other quantitative feedbacks, such as average readings and time above WHO levels (Fig. 3 quote 7). This caused some to reflect on and change their smoking in the home. In most cases, their prime motivation was to better protect their children from SHS (Fig. 3 quote 8).

Participants also reflected that personalized data made the dangers of SHS more real to them than if they had simply heard or read about it. For some, having personalized data was the ‘proof’ they needed to reflect on and change their smoking behaviour (Fig. 3 quote 9). This motivated others to reflect on their smoking more generally, including three women who said that they had made a quit attempt and others who had reduced their smoking.

### Opportunity

Whilst women often displayed increased capability and/or motivation to create a smoke-free home, their opportunities to do so were limited. Only seven reported having the opportunity to change their own or household members’ smoking behaviour. Five participants reported creating a smoke-free home, though only three had PM_2.5_ measurements that confirmed this, as two participants reported creating a smoke-free home after their last PM_2.5_ measurement was taken at the 6-month follow-up visit by their FS worker. Physical environmental factors and/or social relationships and contexts were described as constraining whether, and to what extent, changes were made and sustained. Sometimes this arose from being a single parent, unable to leave young, mobile children to go outside to smoke (Fig. 4 quote 1). This was illustrated by Participant 8 who moved between the second and third measurements, from a third floor flat with no easy access to outdoor space, to a flat with a veranda where she could now smoke as ‘*even when it’s raining you have got a shelter!’.*

Several women reported challenges involving household members continuing to smoke indoors while they were out of the house or asleep, which undermined their own behavioural change (Fig. 4 quotes 2 & 3). Participants dealt with often unstable living circumstances, resulting from relationship breakdowns, drug addiction, homelessness, partners in and out of prison, children in social care, re-housing and estranged families. Fluid household membership was common, with households’ smoking status often varying during the study. This posed extra challenges and reduced opportunities to create smoke-free environments, making behaviour change difficult to even contemplate (Fig. 4 quote 4).

## Discussion

### Main findings of this study

The FS2SF intervention appeared to increase women’s capability to change their smoking behaviour in the home. For some, the shock associated with learning their PM2.5 readings were higher than anticipated, and the personalized nature of the feedback increased their motivation to act. In most cases, the underlying motivation was to protect their children’s health. Our findings also indicate that when social and physical opportunities are restricted, often from limited control over their home environment, changing smoking behaviour in the home becomes less feasible using this intervention approach. This fits with the quantitative findings, which showed no change in household SHS concentrations after the intervention.^20^ This highlights the key limitation of the intervention–it’s inability to fully address the constraints on ‘choice’ associated with challenging and changing life circumstances, women’s often limited power to influence the behaviour of significant others, and the increasing mobility of children in their early years, which are important obstacles to taking smoking outside. Encouraging women who experience intersecting dimensions of disadvantage to be responsible for making ‘choices’ about their lives, and those of their families/children, and attempting to empower them to do so, has limited impact if the socio-economic conditions they live in are insufficient to facilitate change.

### What is already known on this topic

There has been increasing awareness of the need for health-promotion approaches that recognize the gendered nature of health issues and develop gender accommodating and transformative approaches that empower women rather than exploit gender roles and norms.^23^^,^^24^^,^^25^ While adopting an asset-based approach, the FS2SF intervention placed the onus on women to convince partners/other family members to assist in creating a smoke-free home. This approach reflects smoke-free homes literature, which is dominated by interventions focusing on mothers.^6^ However, research on the barriers and facilitators to changing smoking behaviour in these settings has revealed gender power imbalances and women’s limited agency in changing the smoking behaviour of other adults in their household.^6^

### What this study adds

Smoke-free homes interventions need to acknowledge and accommodate the precarious and complex nature of vulnerable, disadvantaged women’s lives, which makes creating smoke-free homes difficult. In the FS2SF study, the shock that most participants experienced when their personalized feedback displayed higher PM_2.5_ readings than expected caused some to reflect on and change their smoking behaviour, taking their smoking outside so as to better protect their children from SHS exposure in the home. However, some participants were unable to fully protect their children from SHS exposure in the home, as a result of housing and/or childcare constraints, and household relations. Future research and policy initiatives must ensure that disadvantaged women’s sense of hope, self-efficacy and well-being are not unduly compromised as a result of interventions designed to encourage efforts to create a smoke-free home.^26^ Using NRT for temporary abstinence or reducing smoking in the home could address some of these complexities, eliminating parents’ need to go outside whilst caring for young children.^15^^,^^16^ More fundamentally though, the FS2SF findings support the Kuala Lumpur Smoke-Free Homes Charter’s^27^ call for smoke-free home interventions to be delivered at a household level rather than only targeting mothers. Developing smoke-free home interventions that work with men and other family members, framing household smoking as a collective responsibility rather than tasking women as mothers and wives to persuade others to take their smoking outside, would address gender-specific issues. However, the feasibility of health professional delivery in ‘real world’ settings remains unclear.^20^

### Limitations of this study

This qualitative study used a purposive sample and therefore the findings may not be generalizable to all study participants. Women who perceived that they had made positive changes may have been more likely to be interviewed and give more positive accounts than other FS2SF participants. Participants’ accounts may have been influenced by social desirability, especially given the shock that some mothers described in response to unexpectedly high SHS levels, and given that FS workers were generally present when the interviews took place.

## Notes

**R. O’Donnell,** Dr.

**A. Amos,** Professor

**S.W. Turner,** Professor

**L. Adams,** Ms.

**T. Henderson,** Ms.

**S. Lyttle,** Ms.

**S. Mitchell,** Ms.

**S. Semple,** Dr.

## References

[1] Cook DG, Strachan DP. Health effects of passive smoking: summary of effects of parental smoking on the respiratory health of children and implications for research. Thorax 1999;54:457–66. 10.1136/thx.54.4.357 PMC1745458 10092699

[2] Royal College of Physicians. Passive Smoking and Children. A Report by the Tobacco Advisory Group. London: Royal College of Physicians, 2010.

[3] Office on Smoking and Health (US). The Health Consequences of Involuntary Exposure to Tobacco Smoke: A Report of the Surgeon General. Atlanta (GA): Centers for Disease Control and Prevention (US), 2006. 20669524

[4] Jarvis MJ, Feyerabend C. Recent trends in children's exposure to second-hand smoke in England: cotinine evidence from the health survey for England. Addiction 2015;110:1484–92. 26061741 10.1111/add.12962

[5] Scottish Government. The Scottish Health Survey*,* Vol. 1, 2016. Main Report. 2018-08-20*.* https://www.gov.scot/Publications/2017/10/2970 (5 August 2019, date last accessed).

[6] Passey ME, Longman JM, Robinson J et al. Smoke-free homes: what are the barriers, motivators and enablers? A qualitative systematic review and thematic synthesis BMJ Open 2016;6:e010260. 10.1136/bmjopen-2015-010260 PMC4800143 26988351

[7] Rowa-Dewar N, Amos A. Disadvantaged parents’ engagement with a national secondhand smoke in the home mass media campaign: a qualitative study. Int J Environ Res Public Health 2016;13:901. 10.3390/ijerph13090901 PMC5036734 27618085

[8] Rowa-Dewar N, Lumsdaine C, Amos A. Protecting children from smoke exposure in disadvantaged homes. Nicotine Tob Res 2015;17:496–501. 25762761 10.1093/ntr/ntu217

[9] Robinson J, Kirkcaldy A. Disadvantaged mothers, young children and smoking in the home: mothers’ use of space within their homes. Health Place 2007;13:894–903. 17499542 10.1016/j.healthplace.2007.03.001

[10] Alwan N, Siddiqi K, Thomson H et al. Can a community-based ‘smoke-free homes’ intervention persuade families to apply smoking restrictions at home? J Public Health 2010;33:48–54. 10.1093/pubmed/fdq073 20930040

[11] Behbod B, Sharma M, Baxi R et al. Family and carer smoking control programmes for reducing children's exposure to environmental tobacco smoke. Cochrane Database Syst Rev 2018;(Issue 1): Art. No.: CD001746. 10.1002/14651858.CD001746.pub4 PMC6491082 29383710

[12] Rosen LJ, Myers V, Winickoff JP, Knott J. Effectiveness of interventions to reduce tobacco smoke pollution in homes: a systematic review and meta-analysis. Int J Environ Res Public Health 2015;12:16043–59. 26694440 10.3390/ijerph121215038 PMC4690974

[13] Wilson I, Mills LM, Ritchie D et al. REFRESH—reducing families' exposure to second-hand smoke in the home: a feasibility study. Tob Control 2013;22:5. 10.1136/tobaccocontrol-2011-050212 22615325

[14] Wilson I, Ritchie D, Amos A et al. ‘I’m not doing this for me’: mothers’ accounts of creating smoke-free homes. Health Educ Res 2013;28:165. 22843328 10.1093/her/cys082

[15] Marsh J, McNeill A, Lewis S et al. Protecting children from secondhand smoke: a mixed-methods feasibility study of a novel smoke-free home intervention. Pilot Feasibility Stud 2016;2:53. 27965870 10.1186/s40814-016-0094-7 PMC5153871

[16] Ratschen E, Thorley R, Jones L et al. A randomised controlled trial of a complex intervention to reduce children’s exposure to secondhand smoke in the home. Tob Control 2018;27:155–62. 28432210 10.1136/tobaccocontrol-2016-053279 PMC5870442

[17] Cancer Research UK. *Cutting down: the reality of budget cuts to local tobacco control**.* A Survey of Tobacco Control Leads in Local Authorities in England. London; CRUK. http://www.cancerresearchuk.org/sites/default/files/local_authority_survey_2016_report_cruk_finalfinal.pdf (05 August 2019, date last accessed).

[18] Morgan A, Ziglio E. Revitalising the evidence base for public health: an assets model. Promot Educ 2007;14:17–22. 17685075 10.1177/10253823070140020701x

[19] Glasgow Centre for Population Health. Towards Asset- Based Health and Care Services*.* 2014. https://www.gcph.co.uk/assets/0000/4200/BPCS13_Towards_asset-based_health_and_care_services_FINAL.pdf (01 August 2019, date last accessed).

[20] Semple S, Turner S, O’Donnell R et al. Using air-quality feedback to encourage disadvantaged parents to create a smoke-free home: results from a randomised control trial. Environ Int 2018;120:104–10. 30076982 10.1016/j.envint.2018.07.039

[21] Neale J. Iterative categorisation (IC): a systematic technique for analysing qualitative data. Addiction 2016;111:1096–106. 26806155 10.1111/add.13314 PMC5069594

[22] Michie S, van Stralen MM, West R. The behaviour change wheel: a new method for characterising and designing behaviour change interventions. Implement Sci 2011;6:42. 21513547 10.1186/1748-5908-6-42 PMC3096582

[23] Amos A, Greaves L, Nichter M, Bloch M. Women and tobacco: a call for including gender in tobacco control research, policy and practice. Tob Control 2012;21:236–43. 22166266 10.1136/tobaccocontrol-2011-050280

[24] Pederson A, Poole N, Greaves L et al. Envisioning Gender-Transformative Health Promotion, Ch1. In: Greaves L, Pederson A, Poole N (eds). Making it Better – Gender Transformative Health Promotion, Vol. 11. Toronto: Women’s Press, 2014, 792–803.

[25] Greaves L. Can tobacco control be transformative? Reducing gender inequity and tobacco use among vulnerable populations. Int J Environ Res Public Health 2014;11:792–803. 24402065 10.3390/ijerph110100792 PMC3924474

[26] Greaves LJ, Hemsing NJ. Sex, gender and second hand smoke policies: implications for disadvantaged women. Am J Prev Med 2009;37(2Suppl):S131–7. 19591752 10.1016/j.amepre.2009.05.012

[27] Semple S, Abidin E, Amos A, Hashim Z, Siddiqi K, Ismail N, on behalf of the participants of the Smoke-Free Homes Workshop. (Kuala Lumpur, 7–9 May 2018). The *Kuala Lumpur Charter on Smoke-free Homes*. https://blogs.bmj.com/tc/2018/06/25/the-kuala-lumpur-charter-on-smoke-free-homes (05 August 2019, date last accessed).

